# Unbearable suffering: mental health consequences of the October 2023 Israeli military assault on the Gaza Strip

**DOI:** 10.1136/bmjgh-2023-014835

**Published:** 2024-09-30

**Authors:** Hanna Kienzler, Gwyn Daniel, Weeam Hammoudeh, Rana Nashashibi, Yasser Abu-Jamei, Rita Giacaman

**Affiliations:** 1 Global Health and Social Medicine, King's College London, London, UK; 2 Consultant Systemic Psychotherapist and Trainer, Oxford, UK; 3 Institute of Community and Public Health, Birzeit University, Bir Zayt, West Bank, Palestine, State of; 4 Palestinian Counseling Centre, Ramallah, Palestine, State of; 5 Gaza Community Mental Health Programme, Gaza, Palestine, State of

**Keywords:** Global Health, Mental Health & Psychiatry, Public Health

**SUMMARY BOX**

- The onslaught on Gaza is leading to catastrophic psychological consequences which will not be limited to the short term and to those directly affected, but will have long-term, intergenerational effects.
- This commentary argues that to understand trauma responses among Gazans, we need to go beyond individual illness and, instead, link the biomedical sphere with the political sphere through the concept of social suffering and, thereby, expose the socio-political conditions of life and the collective trauma-inducing nature of the Israeli military occupation and repression.
- Addressing these complex trauma responses requires approaches that bring together the political, social and personal-level components of mental health with a focus on three factors: safety and allyship; the right to agency and acknowledgement, accountability and reparations.

Current media attention has mainly focused on the horrendous consequences of Israel’s onslaught on the people of the Gaza Strip, its rates of death, serious injury, on people’s struggle for physical survival and on the destruction of infrastructure. However, the integral mental health consequences of this violent onslaught also need to be highlighted, especially as decades of research have determined and verified that violent conflicts cause and gravely exacerbate overall rates of mental disorders.^1^

The profound suffering experienced by the people of the Gaza Strip is unfolding in the context of the deadliest military assault by Israel against Palestinians since the 1948 *Nakba*.^2^ We are witnessing the wholescale destruction of homes, hospitals, schools, universities, mosques, churches and the vital infrastructure required to sustain peoples’ social and economic worlds.^3^,^7^ South Africa’s landmark genocide case against Israel at the International Court of Justice (backed by United Nations experts, human rights organisations and scholars) outlines that the mass killings of Palestinians, their displacement, the bodily and mental harm inflicted, the destruction of the health system, the intentional blockade of food, water and medicine and the prevention of Palestinian births constitute genocidal crimes against humanity.^8^

Alongside the systematic destruction of Gaza’s health system with over 1000 documented attacks on healthcare (ie, incidents where healthcare facilities were damaged, health workers killed and health workers arrested),^9^ the mental health system has totally collapsed. All six Community Mental Health Centres ran out of medications and were forced to close, and the only existing inpatient psychiatric hospital was shelled and can no longer function.^10^ No therapeutic support is available to people with severe mental illness and those who are experiencing multiple traumas, grief, vulnerability, uncertainty and helplessness with the likelihood of catastrophic long-term psychological consequences. Children are especially vulnerable to such consequences from their exposure to extreme violence; loss of parents, family members and friends; absence of any safety; the trauma of having their limbs amputated; and the extreme hunger and thirst they experience daily.^11^ Reports reveal that children are experiencing acute trauma responses including frozenness, mutism, convulsions, confusion and loss of bladder control.^12^ Adults and children alike face extreme anxiety, fear, constant worry about their own safety and that of their loved ones. Even with a cessation of bombing, nightmares and disturbing memories, insomnia, separation anxiety and the bottling up of emotions are likely consequences.^13^ Furthermore, caregivers experience acute stress. Parents feel that their parenting capacities are diminished because the inability to protect one’s own children is such a profoundly debilitating experience. A Save the Children staff member in the Gaza Strip, himself a father, said: ‘Death is everywhere. My children look into my eyes every day, they are searching for answers. I have no answers for them’.^14^ In the case of orphaned children, caregivers and organisations are overwhelmed with extra responsibility amid minimal resources. Many children who lost all family members are supported by the SOS Children’s Village. One of their social workers said in 2023: ‘Every night I wake up (…) terrified, look at my children, and when I see they are still here I feel relieved’. She continued saying: ‘If I die, I wish that all of us die so that no one feels the pain of losing a loved one. I feel that I will need an intensive psychological support when all this (ends)’.^15^

These horrendous experiences must be understood within the context of previous violent assaults. The psychological consequences of this chronic violence and warfare have mainly been elicited through epidemiological research focussing on individual-level responses. For instance, research carried out among Gazan women by Physicians for Human Rights and the Gaza Community Mental Health Programme found that 47.6% showed signs of severe psychological distress and 80.9% demonstrated signs of anxiety.^16^ Other research has found high prevalence of fears; aggression; poor psychological adjustment; personality and behavioural changes; neuroticism; low self-esteem; concentration, attention and memory problems; risk taking; anxiety disorders and mood disorders among others.^17^

Without undermining the importance of such research, we need to go beyond individual illness responses. Giacaman has coined the concept of ‘the invisible wounds inside’ to link the biomedical sphere with the political sphere through the concept of suffering. She, thereby, emphasises socio-political conditions and the collective trauma-inducing nature of the Israeli military occupation and repression.^18^ Furthermore, the ‘invisible wounds inside’ are multidimensional and intergenerational, bringing together past, present and future. For example, the current catastrophe evokes collective traumatic memories of extreme suffering and hardship for Gaza Strip’s citizens whose parents, grandparents or great grandparents experienced the trauma of the *Nakba*. A second *Nakba* through the ethnic cleansing of the Gaza Strip is all too real.

Trauma of this order is not limited to the present and to those directly affected. It has the capacity to create severe intergenerational trauma as evidenced among children of Holocaust survivors, children of Japanese Americans incarcerated during World War II and among children of Cambodian genocide survivors living in the USA. Thus, responding to trauma and, more broadly, the ‘invisible wounds inside’ comes with responsibility beyond the clinical to address their psychological, social and political manifestations—aspects that tend to be obfuscated by illness classifications. This is not only the work of specialised mental health providers. It requires a multilayered response rooted in social justice whereby justice is a crucial prerequisite for healing,^19^ and where those most affected are centrally involved in assessing their needs and developing meaningful and diverse support mechanisms.^20^

At this time, it is essential to be open and realistic about the multiple catastrophic consequences of Israel’s ongoing military onslaught. Israel has strategically targeted communal sources of sustenance and support by wiping out entire extended families and destroying spaces where people bond and provide each other with mutual support and aid such as schools, mosques and churches. The death toll increases so relentlessly, and the destruction of infrastructure is so pervasive that families are no longer able to carry out mourning rituals to two times per day their loved-ones farewell and to receive or extend condolences, making any form of closure extremely difficult. Every effort to intervene, thus, seems minuscule in the face of the destruction of physical and social environments.

What is needed is rebuilding the material, social and inner life worlds to support personal, communal and intergenerational recovery and healing. This argument is illustrated by one of our coauthors, Rana Nashashibi, a counselling psychologist, who stated: ‘Next time I go to Gaza, I won’t go as psychologist. I will go as a builder. What is needed is rebuilding. I’d rather build a house than offer counselling to individuals’

Three factors are essential if healing is ever to take place at individual and community levels in the Gaza Strip:

1. *Safety and allyship*. Safety through the immediate and permanent cessation of warfare and violence is paramount. Only then can Gazans rebuild the violently obliterated infrastructures, homes, schools, places of worship and healthcare services and begin to work towards recovery. Gazans also especially need the capacity to rebuild their own lives, human resources and infrastructures rather than being reduced to ‘recipients of aid’. This so often happens in crisis situations when services are developed and delivered by international actors who fail to take local socio-political contexts, culture, language and specific needs into account, thereby hampering or even harming the recovery process in the long-term. This does not mean that there is no role for international groups to play; on the contrary, their financial support, technical expertise and meaningful allyship is invaluable.
2. *Right to agency*. Rebuilding and recovery hinge on people having the right to agency in determining their own future in a world where they are recognised as fully human. This requires a transformation in the global narrative about Gaza from racist tropes that dehumanise Palestinians as ‘terrorists’, ‘human animals’, ‘human shields’ or ‘humanitarian cases’ to a people legitimately resisting injustice and seeking liberation to be able to rebuild their lives materially, socially, and culturally.
3. *Acknowledgement, accountability and reparations*. Rebuilding and recovery require perpetrators of violence to acknowledge the pain and suffering they have caused. This can only happen if they are held accountable for their deeds. Ensuring accountability requires the prosecution of Israel through international courts investigating war crimes and genocide and a demand for reparations. Reparations will be necessary for rebuilding the infrastructure in the Gaza Strip that was destroyed in this war and in previous ones and for the ‘de-development’ endured as a consequence of the long-term blockade. In other contexts, reparations have been shown to be a public health priority as they can afford people a dignified life by reducing the stressors linked to extreme poverty, over the generations.^21^

While we do not claim to know how these factors can be activated in the current political climate, we know that for any of these to have their intended effect depends on ending the war, and a new determination from the international community to demand and promote freedom, justice, sovereignty and self-determination for Palestinians. As highlighted by Article 16 of the Sustainable Development Goals, the promotion of peace and inclusivity also requires building effective, accountable and inclusive institutions at all levels. These are, in any given society, the foundations for living dignified, meaningful and flourishing lives in which hopes, dreams and aspirations feel attainable, and people can achieve mental well-being within their communities and across generations.

## Data Availability

There are no data in this work.
